# Pulmonary infection associated with immune dysfunction is associated with poor prognosis in patients with myelodysplastic syndrome accompanied by *TP53* abnormalities

**DOI:** 10.3389/fonc.2023.1294037

**Published:** 2023-11-30

**Authors:** Yi Chen, Jing Zheng, Yanyan Qiu, Zhengjun Wu, Xiaofeng Luo, Liangfang Zhu, Yong Wu, Yanjuan Lin

**Affiliations:** Fujian Medical University Union Hospital, Fujian Institute of Hematology, Fujian Provincial Key Laboratory on Hematology, Fuzhou, Fujian, China

**Keywords:** myelodysplastic syndrome, tumor protein 53, pulmonary infection, immune dysfunction, prognosis

## Abstract

The aim of this study was to examine the characteristics and prognosis of patients with myelodysplastic syndrome (MDS) accompanied by *TP53* abnormalities and explore potential prognostic factors and treatment responses. This retrospective analysis included 95 patients with MDS and *TP53* abnormalities and 173 patients with MDS without *TP53* abnormalities at the Fujian Medical University Union Hospital between January 2016 and June 2023. Among patients with *TP53* abnormalities, 26 (27.4%) developed AML during the disease course, with a median transformation time of 5.7 months. Complex karyotypes were observed in 73.1% of patients, and the proportions of -5 or del(5q), -7 or del(7q), +8, and -20 or del(20q) were 81.8%, 54.5%, 30.7%, and 25.0%, respectively. These patients exhibited poor survival, with a median overall survival (OS) of 7.3 months, and had 1- and 2-year OS rates of 42.2% and 21.5%, respectively. The complete response rates for azacitidine monotherapy, venetoclax combined with azacitidine, decitabine monotherapy, and decitabine combined with low-dose chemotherapy were 9.1%, 41.7%, 37.5%, and 33.3%, respectively. Long-term survival was similar among the four treatment groups. Patients who underwent allogeneic hematopoietic stem cell transplantation (allo-HSCT) had a median OS of 21.3 months, which trended to be longer than that of patients who did not undergo allo-HSCT (5.6 months; P = 0.1449). Patients with pulmonary infection at diagnosis experienced worse OS than those without pulmonary infection (2.3 months vs. 15.4 months; P < 0.0001). Moreover, 61.9% of patients with pulmonary infection had immune dysfunction, with a ratio of CD4+ to CD8+ T lymphocytes below two. Pulmonary infections and complex karyotypes were independent adverse prognostic factors for OS. In conclusion, *TP53* abnormalities in patients with MDS were frequently accompanied by complex karyotypes, and treatments based on hypomethylating agents or venetoclax have limited efficacy. Pulmonary infections associated with immune dysfunction is associated with poor prognosis.

## 1 Introduction

Since its identification in 1979 and the revelation of its role as a tumor suppressor gene in 1989, tumor protein 53 (*TP53)* has been a hot topic in the field of cancer research (1). *TP53* is the most frequently mutated gene in cancer (2, 3). The frequency of *TP53* mutations is highly variable among the different types and stages of cancers (4). Myelodysplastic syndrome (MDS) is a group of acquired clonal stem cell disorders that is very heterogeneous in its morphology, clinical features, and survival (5). *TP53* mutations are detected in approximately 10%-20% of patients with *de novo* MDS and 30%-40% of patients with therapy-related MDS (6, 7), and *TP53* abnormalities occur in 70–80% of patients with complex karyotypes or a loss of chromosome 17/17p, 5/5q, or 7/7q (8, 9). *TP53* abnormalities in MDS are associated with high-risk disease, rapid transformation to acute myeloid leukemia (AML), resistance to conventional therapies, and poor outcomes (10–13). However, the clinical value of *TP53* abnormalities in patients with MDS has not been fully investigated.

Despite being one of the most studied genes since its discovery, *TP53* is considered “undruggable.” Therefore, *TP53*-mutated MDS remains a long-standing therapeutic challenge, with a median survival of only 5–10 months, irrespective of the therapies administered (14). Hypomethylating agents (HMAs) are the current standard treatment for newly diagnosed high-risk MDS and offer an overall response rate (ORR) of 17%–77% in patients with *TP53*-mutated MDS, with International Working Group complete response (CR) in 10–25% of patients and a median overall survival (OS) of 8.2–12.4 months (15, 16). Venetoclax, a selective small-molecule B-cell lymphoma 2 inhibitor, is a promising agent for the treatment of myeloid malignancies. A retrospective study showed an ORR of 57.2% and a median OS of 14 months in patients with refractory/relapsed (R/R) MDS treated with venetoclax combined with HMAs (17). Recent clinical trials have shown that venetoclax with azacitidine is effective in patients with high-risk R/R MDS, provides clinically meaningful benefits, and improves OS (18, 19). Presently, treatments based on HMAs or venetoclax are the main choices for high-risk MDS; however, the efficacy of these regimens in the treatment of MDS with *TP53* abnormalities needs to be further clarified.

Additionally, immune dysfunction associated with *TP53* mutation has recently been observed in patients with MDS. Sallman et al. concluded that the microenvironment of *TP53*-mutated MDS has an immune-privileged, evasive phenotype that may be a primary driver of poor outcomes and suggested that immunomodulatory therapeutic strategies may improve survival in this molecularly defined subpopulation (20). Recently, novel immunotherapeutic approaches have been developed for *TP53*-mutated MDS and AML and have demonstrated promising results (21, 22). Currently, few studies have focused on the relationship between immune dysfunction and infection in MDS with *TP53* abnormalities.

The aim of this study was to examine the characteristics and prognosis of MDS patients with *TP53* abnormalities and explore potential prognostic factors for OS in patients receiving HMA- or venetoclax-based treatments. We also evaluated the prognostic value of pulmonary infections associated with immune dysfunction in this cohort.

## 2 Methods

### 2.1 Patients

This single-center retrospective study included 95 consecutive patients diagnosed with MDS and *TP53* abnormalities and 173 patients diagnosed with MDS without *TP53* abnormalities between January 2016 and June 2023. This study was approved by the Ethics Committee of Fujian Medical University Union Hospital (2023KY154), and all patients provided written informed consent for treatment.

### 2.2 Definition and classification of MDS

MDS was diagnosed and classified according to the 5th edition of the World Health Organization Classification of Haematolymphoid Tumours (23).

### 2.3 Definition of complex karyotype and assessment of *TP53* mutations

Complex karyotype was defined as having three or more chromosomal abnormalities. *TP53* gene mutation assessment was performed by Kangsheng Global Medical Technology Co., Ltd. gDNA was extracted from the patient’s bone marrow (BM) sample, then amplified by multiplex polymerase chain reaction (PCR). The detection of a whole exon of the *TP53* gene was performed by an illumina-based NEXTSeq550 sequencer.

### 2.4 Definition of pulmonary infection and ratio of CD4+ to CD8+ lymphocyte

The diagnosis of pulmonary infection was based on the patient’s clinical symptoms and signs, in combination with a computed tomography (CT) scan of the lung and pathogenic indicators, such as sputum culture and nucleic acid testing of pathogens. Pleural effusion was diagnosed according to a CT scan of the lung.

The samples for analysis of T cell populations were acquired from patients’ peripheral blood and examined by flow cytometry using the BD FACS Canto II. CD45/SSC, CD3/SSC, and CD19/SSC were used for gating. B lymphocytes were labeled with anti-human CD10/FITC, CD20/PE, CD19/PE-Cy7, CD20/APC-Cy7, and CD45/PerCP; T and natural killer (NK) lymphocytes were labeled with anti-human CD3/FITC, CD16/PE, CD56/PE, CD45/PerCP, CD8/PE-Cy7, and CD4/APC-Cy7. All the antibodies used were acquired from BD Biosciences. The ratio of CD4+ to CD8+ lymphocytes below two was classified as immune dysfunction.

### 2.5 Treatment protocols

Among the patients with MDS and TP53 abnormalities, fifty-eight patients received HMA-based therapies, including 18 who received azacitidine and 40 who received decitabine. Twelve patients were administered a combination of venetoclax and azacitidine. Ten patients underwent allogeneic hematopoietic stem cell transplantation (allo-HSCT). Details of the initial treatment protocols for these patients are summarized in **Table 1**.

**Table 1:** Initial treatment protocols.

Protocols	No.	Regimens
HMA-based therapies	58	
Azacitidine-based therapies	18	
Azacitidine monotherapy	15	4-week cycle: azacitidine: 75 mg/m^2^, subcutaneous injection once a day from days 1 to 7.
Azacitidine + Low dose chemotherapy	3	4-week cycle: azacitidine: 75 mg/m^2^, subcutaneous injection once a day from days 1 to 7; aclacinomycin, 10 mg per day, intravenous injection once a day from days 3 to 6; cytarabine: 10 mg/m^2^, intravenous injection twice a day from days 3 to 9; G-CSF: 5 mg/kg, subcutaneous injection once a day from day 0 to granulocyte deficiency recovery.
Decitabine-based therapies	40	
Decitabine monotherapy	29	4-week cycle: decitabine: 20 mg/m^2^, intravenous injection once a day from days 1 to 5.
Decitabine + low dose chemotherapy	11	4-week cycle: decitabine: 20 mg/m^2^, intravenous injection once a day from days 1 to 5; aclacinomycin, 10 mg per day, intravenous injection once a day from days 3 to 6; cytarabine: 10 mg/m^2^, intravenous injection twice a day from days 3 to 9; G-CSF: 5 mg/kg, subcutaneous injection once a day from day 0 to granulocyte deficiency recovery.
Venetoclax + azacitidine	12	4-week cycle: azacitidine: 75 mg/m^2^, subcutaneous injection once a day from days 1 to 7; venetoclax: 100 mg day 1, 200 mg day 2, 400 mg from days 3 to 28, oral administration once a day.
Other treatments	25	
All-trans retinoic acid ± danazol ± thalidomide	6	All-trans retinoic acid: 20 mg twice a day, oral administration; danazol: 200 mg twice a day, oral administration; thalidomide: 75 mg once a day, oral administration.
Allogeneic hematopoietic stem cell transplantation	2	Relatively half-matched hematopoietic stem cell transplantation
Intensive hemotherapy	2	4-week cycle: idarubicin: 10 mg/m^2^, intravenous injection once a day from days 1 to 3; cytarabine: 100 mg/m^2^, intravenous injection once a day from days 1 to 7.
Best supportive care	15	Anti-infection, cytokine, and blood transfusion therapy.

HMA, hypomethylating agent; G-CSF, granulocyte colony-stimulating factor.

### 2.6 Definition of response and outcomes

The therapeutic effects were evaluated according to the revised International Working Group 2023 response criteria for high-risk MDS (24). Peripheral blood counts were obtained for each cycle before treatment. BM aspiration and biopsy were performed every one or two cycles for response assessment. The ORR included CR and partial remission (PR) rates. Duration of response (DOR) was calculated as the time from CR or PR to progression or relapse, and OS was calculated from diagnosis to death or the last follow-up visit.

### 2.7 Statistical analysis

Differences between patient subgroups were analyzed using the chi-square test, *t*-test, or nonparametric test, as appropriate. Survival analysis was performed using the Kaplan–Meier method, and survival curves were compared using the log-rank test. The impact of prognostic factors such as sex, age, cytogenetic abnormalities, and other parameters on OS was analyzed using the Cox regression model. Parameters that had significant impact on OS in the univariate analysis were further included in the multivariate analysis, which was performed with the Cox regression model. All data were analyzed using GraphPad Prism 8.0 software (GraphPad Software, San Diego, CA, USA), the Statistical Package for the Social Sciences software (SPSS version 21.0; IBM Corp., Armonk, NY, USA), and R software (Version 4.03, R Project for Statistical Computing, Vienna, Austria). Statistical significance was set at P < 0.05.

## 3 Results

### 3.1 Clinical characteristics

Among the 95 patients with *TP53* abnormalities, 90 (94.7%) had *TP53* mutations and 5 (5.3%) had *TP53* deletions. The group consisted of 63 male (66.3%) and 32 female (33.7%) patients, with a male-to-female ratio of 1.97:1. Patients’ ages ranged from 15 to 87 years, with a median age of 63 years. Based on morphology, 19 patients (20.0%) had MDS with low blasts, 27 (28.4%) had increased blasts-1 (IB-1), and 49 (51.6%) had increased blasts-2 (IB-2). The median white blood cell count, neutrophil count, hemoglobin concentration, and platelet count were 2.58 × 10 (9)/L, 0.99 × 10 (9)/L, 63 g/L, and 37 × 10 (9)/L, respectively. Fifteen patients (15.8%) had myelofibrosis at the time of diagnosis, as detected by BM biopsy. Chromosomal karyotypes were available for 93 patients, and complex karyotypes were observed in 68 patients (73.1%). Among the 88 patients with fluorescence *in situ* hybridization results, 81.8%, 54.5%, 30.7%, and 25.0% had -5 or del(5q), -7 or del(7q), +8, and -20 or del(20), respectively. *DNMT3A*, *TET2*, *ASXL1*, *SF3B1*, and *ZRSR* mutations were observed in 12 (12.6%), 11 (11.6%), 8 (8.4%), 5 (5.3%), and 4 (4.2%) patients, respectively. Fourteen patients (14.7%) had a history of solid tumors, and 10 (10.5%) had a history of pulmonary tuberculosis. Twenty-six patients (27.4%) developed AML during the disease course, with a median transformation time of 5.7 months. Pulmonary infection was diagnosed in 41 patients (43.2%), and pleural effusion was observed in 36 of the 41 (87.8%) patients with pulmonary infection at the time of diagnosis. The most common microorganisms identified were *Escherichia coli*, *Klebsiella pneumoniae*, and *Candida albicans*. The median proportion of CD4+ lymphocyte and CD8+ lymphocyte were 38.83% (4.02% - 68.42%) and 21.15% (6.73% - 44.8%), respectively. The median CD4+ lymphocyte count in patients with pulmonary infection was 0.312 × 10 (9)/L, which was lower than that of 0.482 × 10 (9)/L in patients without pulmonary infection (P = 0.0941); CD8+ lymphocyte counts were similar between the two groups. Details about the patient characteristics are summarized in **Table 2**.

**Table 2:** Patients characteristics of patients with *TP53* abnormalities.

Parameters	
Median age, y (range)	63 (15-87)
Male sex, n (%)	63 (66.3)
Morphological diagnosis, n (%)	
MDS with low blasts	18 (18.7)
IB-1	29 (30.2)
IB-2	49 (51.1)
Median WBC, 10^9/L (range)	2.58 (0.68-9.69)
Median neutrophil, 10^9/L (range)	0.99 (0.07-6.67)
Median HB, g/dL (range)	6.3 (2.8-11.7)
Median PLT, 10^9/L (range)	37 (0-177)
Median CD4+ lymphocyte count, 10^9/L (range)	0.397 (0.005-1.205)
Median CD8+ lymphocyte count, 10^9/L (range)	0.265 (0.001-0.941)
Median CD4+ lymphocyte proportion, % (range)	38.83 (4.02-68.42)
Median CD8+ lymphocyte proportion, % (range)	21.15 (6.73-44.8)
Ratio of CD4+ / CD8+ lymphocyte proportion	1.53 (0.13-6.01)
*TP53* status, n (%)	
mutation	90 (94.7)
deletion	5 (5.3)
Cytogenetics abnormalities, n (%)	
Complex karyotype	68/93 (73.1)
-5 or del(5q)	72/88 (81.8)
-7 or del(7q)	48/88 (54.5)
+8	27/88 (30.7)
-20 or del(20q)	22/88 (25.0)
Co-mutated genes, n (%)	
*DNMT3A*	12 (12.6)
*TET2*	11 (11.6)
*ASXL1*	8 (8.4)
*ZRSR*	5 (5.3)
*SF3B1*	4 (4.2)
Solid tumor history, n (%)	14 (14.7)
Pulmonary tuberculosis history, n (%)	10 (10.5)
Myelofibrosis, n (%)	15 (15.8)
Pulmonary infection	41 (43.2)
Pleural effusion	36 (37.9)
Secondary AML, n (%)	26 (27.4)
Allo-HSCT	10 (10.5)

MDS, myelodysplastic syndromes; IB, increased blasts; WBC, white blood cell; HB, hemoglobin; PLT, platelet; AML, acute myeloid leukemia; allo-HSCT, allogeneic hematopoietic stem cell transplantation.

### 3.2 Treatment outcomes

Seventy patients (73.9%) who received HMA- or venetoclax-based treatments were included in the efficacy analysis. Among the patients treated with HMA-based therapies, 25.9% achieved CR, with an ORR of 31.5%. The treatment-related mortality rate was 15.5%, and no significant difference was observed between the azacitidine and decitabine groups (P > 0.9999). The CR rate of azacitidine monotherapy was only 9.1%, which was lower than those observed for decitabine monotherapy (37.5%; P = 0.1197) and decitabine combined with low-dose chemotherapy (33.3%; P = 0.5147). Patients receiving venetoclax combined with azacitidine achieved a CR rate of 41.7%, which was superior to that of azacitidine monotherapy (P = 0.1550) and similar to that of decitabine-based treatment (P > 0.9999). Importantly, the median DOR was only 4.4 months in the venetoclax and azacitidine treatment group, which was significantly inferior to that in the decitabine monotherapy treatment group (11.2 months; P = 0.0093). The response rate of patients with *TP53* abnormalities was lower than those without *TP53* abnormalities when treating with HMA- or venetoclax-based therapies (**Table 3**).

**Table 3:** Treatment response of patients with MDS with or without *TP53* abnormalities.

Treatment protocols	Mortality (n, %)	CR (n, %)	ORR (n, %)	Mortality (n, %)	CR (n, %)	ORR (n, %)	P1-value	P2-value	P3-value
	With *TP53* abnormalities			Without *TP53* abnormalities					
HMAs-based therapies	9/58 (15.5)	14/54 (25.9)	17/54 (31.5)	14/148 (9.5)	51/134 (38.1)	83/134 (61.9)	0.2251	0.1293	0.0002
DEC-based therapies	6/40 (15.0)	11/30 (36.7)	14/30 (46.7)	11/116 (9.5)	39/105 (37.1)	65/105 (61.9)	0.3795	>0.9999	0.1470
DEC monotherapy	4/29 (13.8)	9/24 (37.5)	12/24 (50.0)	7/84 (8.3)	27/77 (35.1)	47/77 (61.0)	0.4689	0.8126	0.3537
DEC + Low- dose chemotherapy	2/11 (18.2)	2/6 (33.3)	2/6 (33.3)	4/32 (12.5)	12/28 (42.9)	18/28 (64.3)	0.6367	>0.9999	0.2022
AZA-based therapies	3/18 (16.7)	3/14 (21.4)	3/14 (21.4)	3/32 (9.4)	12/29 (41.4)	18/29 (62.1)	0.6538	0.3084	0.0217
AZA monotherapy	3/15 (20.0)	1/11 (9.1)	1/11 (9.1)	2/25 (8.0)	9/23 (39.1)	13/23 (56.5)	0.3446	0.1133	0.0110
AZA + Low- dose chemotherapy	0/3 (0)	2/3 (66.6)	2/3 (66.6)	1/7 (14.3)	3/6 (50.0)	5/6 (83.3)	>0.9999	>0.9999	>0.9999
Venetoclax + AZA	0/12 (0)	5/12 (41.7)	5/12 (41.7)	2/25 (8.0)	12/23 (52.2)	16/23 (69.6)	>0.9999	0.7247	0.1534

HMAs, hypomethylating agents; DEC, decitabine; AZA, azacitidine; CR, complete remission; ORR, overall response rate. P1-value: comparison of mortality rate between the two groups. P2-value: comparison of CR rate between the two groups. P3-value: comparison of ORR rate between the two groups.

**Figure 1:**
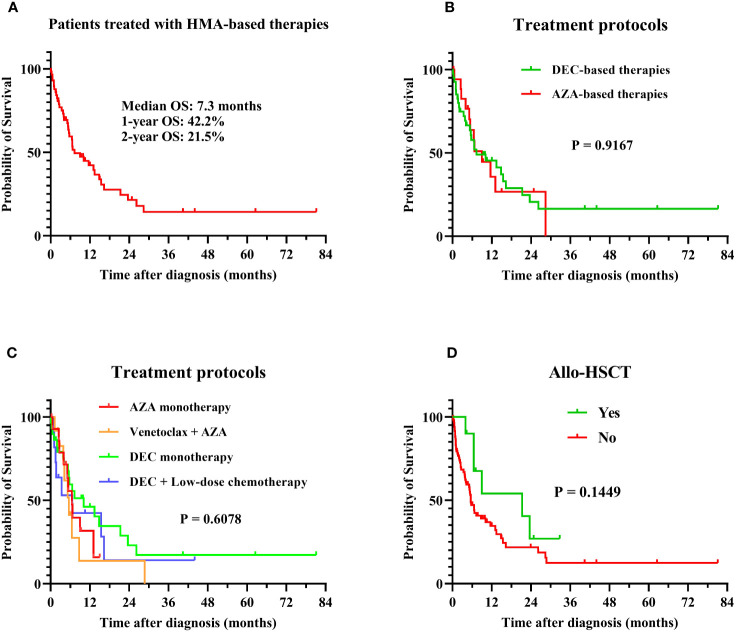
Overall survival in MDS patients with *TP53* abnormalities. Survival analysis was performed using the Kaplan–Meier method, and survival curves were compared using the log-rank test. **(A)** OS of all patients in this study: the median OS was 7.3 months, with 1-year and 2-year OS rates of 42.2% and 21.5%; **(B)** OS of patients treated with HMA-based therapies: the median OS was 7.3 and 9.0 months for AZA-based (N = 18) and DEC-based therapies (N = 40), P = 0.9167; **(C)** OS of patients treated with different regimens: the median OS was 6.5, 5.6, 10.1, and 6.6 months in azacitidine monotherapy (N = 15), venetoclax combined with azacitidine (N = 12), decitabine monotherapy (N = 29), and decitabine with low-dose chemotherapy (N = 11) treatment groups, respectively (P = 0.6078); **(D)** OS of patients treated with or without allo-HSCT: The median OS was 21.3 months and 5.6 months in patients treated with (N = 12) or without (N = 83) allo-HSCT, P = 0.1449. MDS, myelodysplastic syndrome; TP53, tumor protein 53; HMA, hypomethylating agent; allo-HSCT, allogenic hematopoietic stem cell transplantation; DEC, decitabine; AZA, azacitidine; OS, overall survival.

Patients with MDS accompanied by *TP53* abnormalities had poor long-term survival when treated with HMA-based therapies, with a median OS of 7.3 months, 1-year OS rate of 42.2%, and 2-year OS rate of 21.5% (**Figure 1**). Patients who received azacitidine- or decitabine-based treatment had similar outcomes, with a median OS of 7.3 months and 9.0 months, respectively (P = 0.9167; **Figure 1**). The median OS of patients treated with venetoclax and azacitidine was 5.6 months, which was not superior to that of patients treated with HMA-based regimens (**Figure 1**). Patients who underwent allo-HSCT trended to have favorable survival, with a median OS of 21.3 months, whereas patients who did not undergo allo-HSCT had a median OS of 5.6 months (P = 0.1449; **Figure 1**). Patients who developed AML had an extremely poor prognosis, with a median OS of only 1.8 months, 1-year OS rate of 14.7%, and 2-year OS rate of 7.4%. The long-term survival of patients with *TP53* abnormalities was significantly inferior to those without *TP53* abnormalities when treating with HMA- or venetoclax-based therapies (**Table 4**).

**Table 4:** Long-term survival of patients with MDS with or without TP53 abnormalities.

Treatment protocols	Median OS (Month)	1-year OS (%)	2-year OS (%)	MedianOS (Month)	1-year OS (%)	2-year OS (%)	P-value
	With *TP53* abnormalities			Without *TP53* abnormalities			
HMAs-based therapies	7.3	42.2	21.5	50.7	74.8	58.3	<0.0001
DEC-based therapies	7.3	45.5	20.7	52.0	78.2	65.1	<0.0001
DEC monotherapy	10.1	46.2	23.1	Undefined	77.0	64.4	<0.0001
DEC + Low-dose chemotherapy	6.6	42.4	14.1	52.0	80.3	65.9	0.0033
AZA-based therapies	6.5	29.4	23.5	16.1	66.9	34.0	0.0388
AZA monotherapy	6.5	15.9	NA	15.4	69.8	36.2	0.0027
AZA + Low-dose chemotherapy	28.4	66.7	66.7	17.9	57.1	28.6	0.6902
Venetoclax + AZA	5.6	15	15	Undefined	76.4	76.4	0.0009

HMAs, hypomethylating agents; DEC, decitabine; AZA, azacitidine; OS, overall survival; NA: not available; Undefined: the median OS has not yet been reached by the end of follow-up.

### 3.3 Prognostic factors

**Figure 2:**
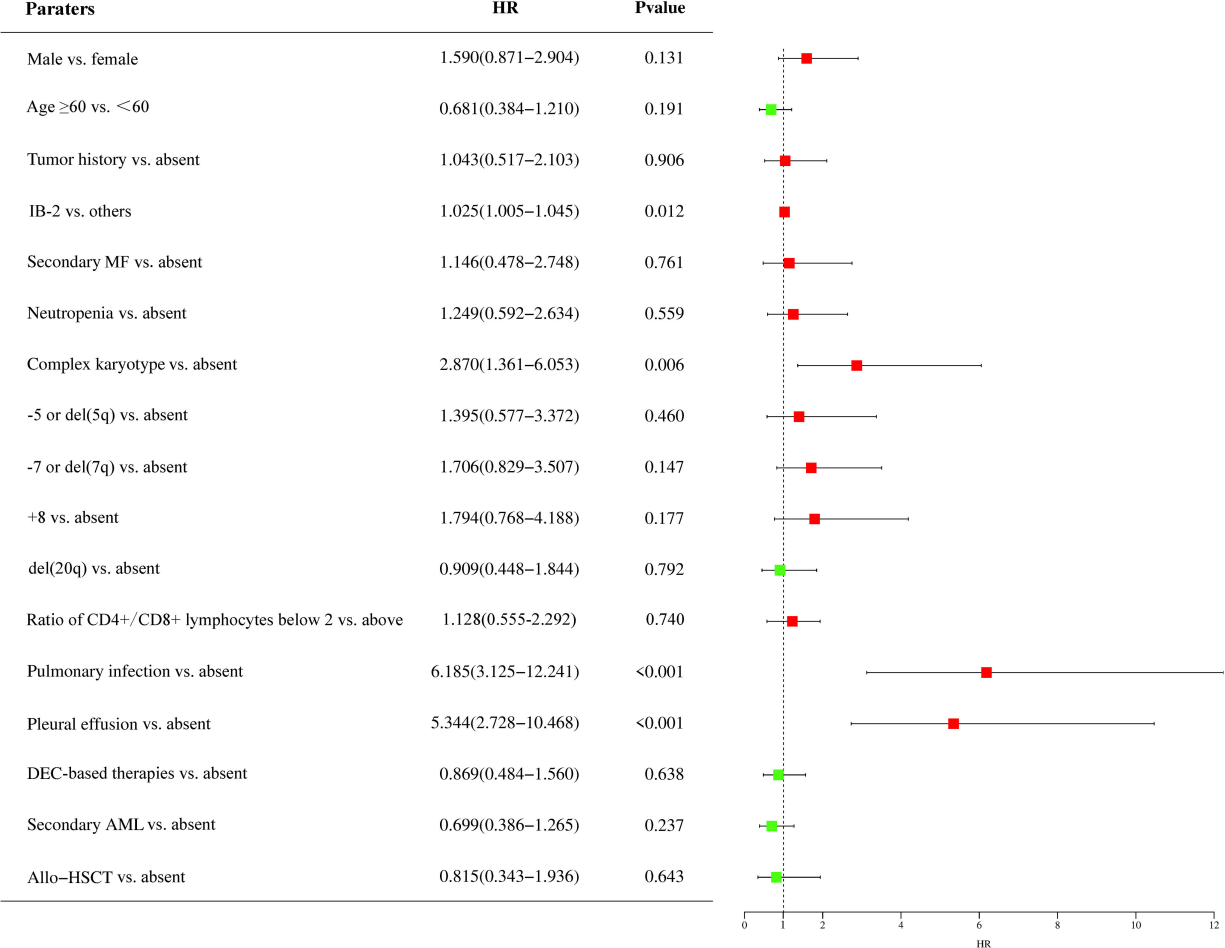
Prognostic factors of patients with MDS accompanied by *TP53* abnormalities. The impact of prognostic factors was analyzed using the Cox regression model, and the results are presented as hazard ratios. HR, hazard ratio; IB-2, increase blast-2; MF, myelofibrosis; DEC, decitabine; AML, acute myeloid leukemia; allo-HSCT, allogeneic hematopoietic stem cell transplantation; MDS, myelodysplastic syndrome; TP53, tumor protein 53.

To identify potential prognostic factors for patients with MDS accompanied by *TP53* abnormalities, we performed univariate and multivariate analyses for OS in patients treated with HMA- or venetoclax-based therapies (**Figure 2**). Univariate analysis identified that IB-2 (hazard ratio [HR] = 1.025, 95% CI: 1.005–1.045, P = 0.012), complex karyotype (HR = 2.870, 95% CI: 1.361–6.053, P = 0.006), pulmonary infection (HR = 6.185, 95% CI: 3.125–12.241, P < 0.001), and pleural effusion (HR = 5.344, 95% CI: 2.728–10.468, P < 0.001) at the time of diagnosis were significant adverse prognostic factors. Additionally, male sex, -7 or del(7q), and +8 aberrations had a trend toward adverse effects on OS, whereas decitabine-based therapies and allo-HSCT had a trend toward favorable effects on OS. We included the IB-2, complex karyotype, pulmonary infection, and pleural effusion in the multivariate analysis and found that complex chromosomal karyotypes (HR = 2.493, 95% CI: 1.164–5.338, P = 0.019) and pulmonary infections (HR = 7.666, 95% CI: 1.605–36.608, P = 0.011) were significant independent adverse prognostic factors for OS.

Forty-one patients (43.2%) had pulmonary infection at the time of diagnosis, and these patients had a worse prognosis than those without pulmonary infection (median OS, 2.3 months vs. 15.4 months; P < 0.0001). The median neutrophil counts were 1.24 (0.13-6.67) × 10 (9)/L and 0.99 (0.07-5.23) × 10 (9)/L in patients with and without pulmonary infection, respectively (P = 0.6377). Pleural effusion was observed in 36 of 41 (87.8%) patients with pulmonary infection. Additionally, myeloid blasts were detected by flow cytometry in some patients with massive pleural effusion, indicating that MDS with *TP53* abnormalities may present with extramedullary invasion. A T cell subpopulation analysis was performed in 21 patients with pulmonary infection, and 13 (61.9%) had a ratio of CD4+ to CD8+ T lymphocytes below two, which revealed immune dysfunction in these patients.

## 4 Discussion

In our study, 25.9% of patients treated with HMA-based regimens achieved CR, with an ORR of 31.5%. Treatment-related mortality rates were similar between the azacitidine and decitabine groups. The CR rate of azacitidine monotherapy was lower than those of venetoclax combined with azacitidine, decitabine monotherapy, and decitabine combined with low-dose chemotherapy. Our data showed that patients with *TP53* abnormalities had poor long-term survival when treated with HMA-based therapies, with median OS, 1-year survival, and 2-year survival that were significantly inferior to those of patients without *TP53* abnormalities in our studies (25). A previous study revealed that venetoclax plus azacitidine improved remission rates, but not DOR or OS, compared with azacitidine alone in patients with *TP53*-mutated MDS with high-risk cytogenetics (26). Our results also confirmed that despite a higher remission rate in the venetoclax with azacitidine and decitabine-based treatment groups compared to the azacitidine monotherapy group, no significant difference in long-term survival was observed. Hence, a higher CR rate did not directly lead to better long-term survival in patients with MDS and *TP53* abnormalities, and subsequent therapies are needed after patients respond to these initial treatment protocols.

MDS is characterized by a high risk for transformation to AML, and approximately 30% of patients with MDS eventually progress to AML as reported in previous studies. In our study, 27.4% of patients developed AML during the disease course with a median transformation time of 5.7 months, which was significantly shorter than that in patients without *TP53* abnormalities in previous studies (27). These patients had an extremely poor prognosis, with a median survival period of only 1.8 months. Therefore, improving the outcomes in this population remains challenging.

Although several drugs have been found to improve disease control in MDS, allo-HSCT remains the only curative treatment (28). However, multiple analyses have shown that patients with *TP53*-mutated MDS and AML harbor an 80–90% higher risk of relapse and death after allo-HSCT than patients with *TP53* wild-type MDS (13, 29–32). The majority of these relapses and deaths following allo-HSCT occur in patients with concomitant chromosome 17 abnormalities or complex karyotypes, leading to multi-hit disease (33). Our results demonstrated that patients with MDS accompanied by *TP53* abnormalities who underwent allo-HSCT trended to show better survival than those who only received HMAs or venetoclax-based treatments, but this advantage was limited and not as good as that in patients without *TP53* abnormalities, as reported previously (34).

Most MDS prognostic scoring systems are based on the BM blast percentage, depth of cytopenia, and cytogenetics, and these three major features have been shown to have a significant impact on the prognosis and risk of AML transformation (35). Recently, *TP53* and other genes mutations were incorporated into the Molecular International Prognostic Scoring System (36). It is well established that a complex chromosomal karyotype is an adverse prognostic factor for MDS, and the absence of *TP53* mutations in patients with complex karyotypes is associated with much better survival than that in patients with *TP53* mutations (37). Our data showed that 73.1% of patients with MDS accompanied by *TP53* abnormalities had complex chromosomal karyotypes, and complex karyotypes were identified as an independent adverse prognostic factor of OS in patients treated with HMA- or venetoclax-based therapies. Thus, *TP53* abnormalities were frequently associated with complex chromosomal karyotypes and dismal prognosis in patients with MDS.

*TP53*-mutated MDS is associated with a risk of severe infection. Neutropenia was previously believed to be the main predisposing factor for this increased risk, but recent studies have discovered immune abnormalities in these patients (38, 39). Previous studies have demonstrated that patients with *TP53*-mutated MDS with infection have worse survival than those without infection (40). In our study, patients with pulmonary infection had extremely poor survival, and pulmonary infection was identified as an independent adverse prognostic factor. Importantly, patients with pulmonary infection had neutrophil counts similar to those in patients without pulmonary infection, and the T cell subpopulation analysis revealed that the majority of these patients had immune dysfunction. Therefore, immune dysfunction might be the main reason for uncontrollable infection and contribute to poor prognosis. However, the specific mechanisms responsible for this phenomenon need to be studied further. Pleural effusion was observed in 87.8% of patients with pulmonary infection, and myeloid blasts were detected by flow cytometry in some patients with massive pleural effusion. Whether patients with pleural infiltration and a percentage of BM blasts less than 20% should be diagnosed with MDS/AML, as proposed by the International Consensus Classification 2022 classification, requires further examination (41).

This study has some limitations that must be considered. Due to the single-center retrospective design and long observation period, some information bias was unavoidable. Additionally, our patients were heterogeneous with several therapeutic regimens used; therefore, the stratification of this population into small groups undermines the statistical power of the study. Because of the limited number of cases and the difficulty in detecting some microorganisms, detecting all pathogens and classifying them according to bacteria, fungi, and virus was impossible, which may have resulted in an additional source of bias. Therefore, prospective studies are still needed to confirm these findings.

In conclusion, *TP53* abnormalities in MDS patients are frequently accompanied by complex karyotypes. Decitabine-based therapies are associated with a higher CR rate but similar long-term survival rates compared to azacitidine-based therapies. Venetoclax plus azacitidine improved the CR rate but did not improve survival due to the short DOR in this population. Pulmonary infection and complex karyotypes are independent adverse prognostic factors for OS. Further studies are required to overcome the poor survival of these patients.

## Data availability statement

The raw data that support the conclusions of this article are available from the corresponding author upon reasonable request.

## Ethics statement

The studies involving humans were approved by Ethical Commission of the Fujian Medical University Union Hospital. The studies were conducted in accordance with the local legislation and institutional requirements. Written informed consent for participation in this study was provided by the participants’ legal guardians/next of kin.

## Author contributions

YC: Conceptualization, Data curation, Writing – original draft. JZ: Data curation, Investigation, Methodology, Writing – original draft. YQ: Formal Analysis, Resources, Validation, Writing – original draft. ZW: Resources, Validation, Writing – original draft. XL: Resources, Software, Validation, Writing – original draft. LZ: Project administration, Resources, Writing – original draft. YW: Conceptualization, Funding acquisition, Writing – review & editing. YL: Conceptualization, Methodology, Writing – review & editing.
